# Awareness and perception of white spot lesions amongst adolescents undergoing orthodontic treatment with fixed appliances: a cross-sectional study of caries preventive adherence

**DOI:** 10.1007/s40368-026-01179-x

**Published:** 2026-02-09

**Authors:** S. P. van Doornik, S. Lietmeijer, Y. Ren, D. J. Manton, P. U. Dijkstra, A. M. Kuijpers-Jagtman

**Affiliations:** 1 Department of Dentistry Orthodontics, University Medical Center Groningen, Groningen, Netherlands; 2 Department of Rehabilitation, University Medical Center Groningen, Groningen, Netherlands; 3 Department of Orthodontics and Dentofacial Orthopedics, University of Bern, Bern, Switzerland; 4 Faculty of Dentistry, Universitas Indonesia, Jakarta, Indonesia

**Keywords:** Orthodontics, White spot lesions, Patient perception, Oral hygiene, Adherence, Fixed appliances

## Abstract

**Background:**

White spot lesions (WSLs) are a common and aesthetically concerning side-effect of orthodontic treatment with fixed appliances. Despite their high prevalence, the impact of adolescent awareness on caries preventive behaviour remains poorly understood.

**Purpose:**

This study investigated associations between WSL awareness, perceived annoyance with WSLs, adherence to caries-related clinical practice guidelines (CPGs), and patient characteristics amongst adolescents receiving orthodontic fixed appliance treatment.

**Methods:**

This cross-sectional study included 393 orthodontic patients aged 12–17 years from ten orthodontic practices. Participants completed an electronic survey assessing WSL awareness, participant characteristics, perceived annoyance (score range: 3–15), and adherence to six CPG behaviours (participants brushed 2 times/day (1), for 2 min (2), using fluoride toothpaste (3), cleaning interdentally (4), yearly dental visits (5), their food and drink intake ≤ 7 times/day (6); score range: 1–6).

**Results:**

Of the 329 valid responses, 38% reported prior awareness of WSLs. These participants had significantly higher annoyance scores compared to those without awareness (median 13) compared to those not aware of WSLs (median 12, *p* = 0.004). However, awareness was not associated with treatment duration or level of CPG adherence. Annoyance was associated with CPG adherence (Spearman’s *ρ* = 0.149, *p* = 0.003). No significant associations were found between annoyance and participant characteristics.

**Conclusions:**

Although prior WSL awareness was associated with greater perceived annoyance, it was not associated with better adherence to caries preventive measures. These findings suggest that whilst adolescents may recognise the aesthetic impact of WSLs, current educational approaches are insufficient to translate concern into patient actions. Additional efforts are needed to promote sustained adherence during orthodontic treatment.

## Introduction

Fixed orthodontic appliances are used commonly to correct dental malocclusions and improve dentofacial aesthetics, thereby enhancing oral health-related quality of life (Silvola et al. 2014; Abreu et al. 2018; Bilici Gecer and Dursun 2024). Despite these clinical benefits, a frequent and well-recognised adverse effect is enamel demineralisation, resulting in white spot lesions (WSLs), which are sub-surface lesions characterised by chalky-white opacity due to mineral loss (Maxfield et al. 2012; Ludovichetti et al. 2025; Yu et al. 2025; Benson et al. 2019). These lesions can develop as early as 4 weeks after orthodontic treatment initiation, particularly in patients with inadequate oral hygiene and a high cariogenic diet (Maxfield et al. 2012; Scheerman et al. 2018; Khoroushi and Kachuie 2017).

According to a recent meta-analysis, the pooled prevalence of WSLs in orthodontic patients is approximately 55% (95% CI: 48–64%) (Hussain et al. 2025). Individual studies, however, report a wide variation ranging from 34% to nearly 97%, depending on diagnostic criteria used, treatment duration, oral hygiene behaviours, dietary habits, and adherence to caries preventive strategies (Hadler-Olsen et al. 2012; Zotti et al. 2016; Tasios et al. 2019). Clinically, WSLs often require remineralisation, resin infiltration, or restorative treatment (Benson et al. 2019). Aesthetically, WSLs are a major source of post-treatment dissatisfaction for orthodontic patients (Huang et al. 2018; Lamorgese et al. 2025).

Patient perceptions of WSLs are shaped by multiple factors, including oral hygiene behaviours, educational level, and severity of malocclusion (Huang et al. 2018; Bilici Gecer and Dursun 2024). Patients with greater oral health knowledge and better hygiene practices tend to be more critical of WSLs and report higher levels of annoyance (Huang et al. 2018). Conversely, patients with severe pre-treatment malocclusion tend to be more tolerant of compromised aesthetic outcomes (Silvola et al. 2014).

Although clinical practice guidelines (CPGs) provide evidence-based strategies for WSL prevention, including oral hygiene instructions, fluoride use, and dietary advice, adherence remains poor (Benson et al. 2019; Ludovichetti et al. 2025; IvorenKruis 2011; NVvO 2022). Many patients report receiving inadequate instructions or have limited recall of the guidance provided (Maxfield et al. 2012; Bilici Gecer and Dursun 2024). A recent study (van Doornik et al. 2025) found that only 22.6% of patients adhered to all six recommendations of the Dutch CPGs for limiting caries risk and preventing WSLs (IvorenKruis 2011; NVvO 2022). Particularly, older adolescents, boys, and those with higher education levels showed lower adherence (van Doornik et al. 2025).

Whilst the clinical burden of WSLs is well described (Hussain et al. 2025; Ludovichetti et al. 2025), the relationship between patients’ awareness of WSLs, their perceived annoyance, and their adherence to CPGs has not been investigated systematically. It is hypothesised that greater awareness may increase perceived annoyance, which in turn could influence caries preventive behaviours, yet empirical evidence remains limited.

Therefore, this study aims to analyse the associations between patients’ awareness of WSLs, perceived annoyance, adherence to caries-related CPGs, treatment duration, and patient characteristics to support more patient-centred caries prevention in orthodontic care.

The primary aim of this study was to explore the association between adolescents’ awareness of WSL and perceived annoyance. Secondary aims were to examine the relationship between annoyance, adherence to caries-related CPGs, treatment duration, and participant characteristics.

## Materials and methods

### Study design and ethical approval

This multi-centre cross-sectional survey was conducted in collaboration with the Orthodontics Research Network Northeast Netherlands (ORN-NN). The present study builds on a previous investigation that examined CPGs adherence in adolescent orthodontic patients (van Doornik et al. 2025). In this secondary analysis, the focus lies specifically on patient awareness of WSLs and their perceived annoyance related to these lesions, using data from the same survey. The Medical Ethics Review Board of the University Medical Center Groningen (UMCG) (METc 2023/014) reviewed and approved the study, concluding that it did not fall under the scope of the Dutch Medical Research Involving Human Subjects Act (WMO). All participants and their legal guardians provided written informed consent before participation. Participation was voluntary; participants could withdraw at any time without penalty. Data were collected anonymously.

### Setting and recruitment

Data were collected between February and April 2023 across nine private orthodontic practices and the Department of Orthodontics at the UMCG, all affiliated with the ORN-NN. Eligible participants were recruited consecutively during routine clinical appointments. Recruitment and on-site survey assistance were performed by trained members of the research team (SvD, SL and RK); orthodontists and dental assistants of the practices were not involved in participant recruitment or data collection*.* The research team also applied inclusion and exclusion criteria on-site. The survey was completed on-site after the orthodontic appointment in the presence of a researcher to answer questions of participants. The survey required approximately 10 min to complete. Practitioners and caregivers were not involved in the recruitment process. All participants completed the survey electronically at the orthodontic practice using a tablet or laptop and a secure, individualised REDCap link (Research Electronic Data Capture; Indiana University, USA) hosted on institutional servers of the UMCG (Harris et al. 2019, 2009).

### Participants

Participants were eligible for inclusion if they were aged between 12 and 17 years, were currently undergoing active treatment with buccal fixed orthodontic appliances in both dental arches from the first permanent molar to the first permanent molar and had been in treatment for at least 3 months. Exclusion criteria were the presence of cleft lip and/or palate or other craniofacial anomalies, intellectual disability requiring specialised care, insufficient literacy in the Dutch language to understand the survey, or prior participation in a pilot study to test the survey. Eligibility was assessed during on-site survey administration.

### Survey instrument development

The survey was developed in close collaboration with faculty experts in orthodontics and cariology at the UMCG, all of whom were PhD-qualified clinicians, to ensure clinical and content validity. The final version comprised three sections: (1) participant characteristics, (2) adherence to CPGs (van Doornik et al. 2025), and (3) awareness and perceived annoyance related to WSLs.

Participant characteristics included age, sex, participant educational level, maternal education level [used as a proxy for household socioeconomic status (SES)], and postal code (as a secondary SES indicator). Educational levels were categorised according to the Dutch educational system as follows: ‘low’ (primary school and lower secondary vocational education), ‘middle’ (senior general secondary education and secondary vocational education), and ‘high’ (pre-university education, university of applied sciences, or university). Treatment duration with buccal fixed appliances was categorised into four intervals to reflect different phases of orthodontic treatment: 0 to < 3 months, 3 to < 6 months, 6 to < 12 months, and ≥ 12 months.

The survey was pilot tested amongst 44 adolescent orthodontic patients at the Department of Orthodontics, UMCG, to assess clarity, comprehensibility, and relevance. Pilot participants met the same inclusion and exclusion criteria as those applied in the primary study. Minor revisions to question phrasing and layout were made based on feedback from both pilot participants and faculty reviewers. The final instrument demonstrated acceptable internal consistency, with a Cronbach’s alpha of 0.716.

### Adherence sum score

Adherence to the CPGs was assessed using six items: (1) brushing teeth at least twice daily, (2) brushing for 2 min, (3) using fluoride toothpaste, (4) using interdental cleaning aids, (5) visiting a dentist or oral hygienist in the past year during orthodontic treatment, and (6) limiting food and beverage consumption to seven or fewer times per day (van Doornik et al. 2025). Each adhered behaviour contributed one point, resulting in a total adherence score ranging from 0 to 6. Higher scores indicated a lower probability of developing WSLs. The use of fluoride toothpaste was assessed dichotomously; fluoride concentration (ppm) was not recorded.

### WSL awareness and annoyance scores

**Fig. 1:**
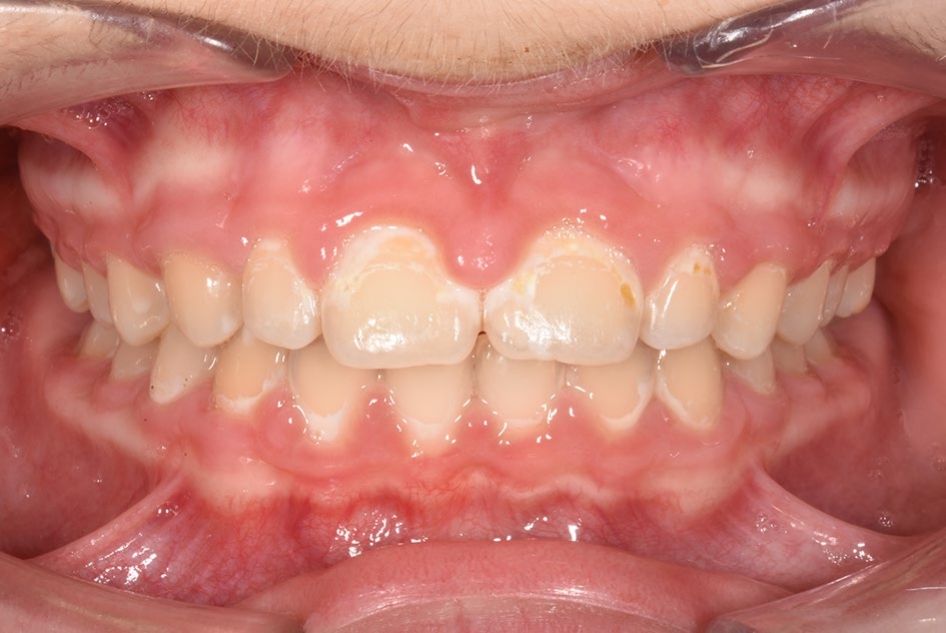
Image shown to the participants illustrating white spot lesions after orthodontic treatment with buccal fixed appliances

To assess WSL awareness, participants were first asked whether they had seen an image of WSLs previously, before completing the survey; no specific time frame was defined. Immediately following this question, a standardised reference image of typical WSLs was presented within the online survey interface to ensure consistent visual understanding across all participants (Fig. 1). Perceived annoyance related to WSLs was assessed using three items, each rated on a 5-point Likert scale ranging from 1 (completely disagree) to 5 (completely agree) (Norman 2010). These items targeted personal and aesthetic aspects of WSL perception and were combined into a single composite score, termed the Annoyance Score (scoring range 3–15). Higher scores reflected greater annoyance regarding the presence of WSLs. In addition, willingness to seek treatment for WSLs and knowledge about the possibility of enamel improvement after fixed appliance removal were each assessed using a 5-point Likert scale (Norman 2010).

### Sample size calculation

The sample size was calculated using G*Power (version 3.1.9.7, Heinrich-Heine-Universität Düsseldorf) for a multivariable linear regression model with five predictors. Assuming a medium-effect size in the annoyance score (f^2^ = 0.15), an alpha level of 0.05, and a power of 0.80, the minimum required sample size was 275 participants. Based on an expected non-response rate of 50% (Wu et al. 2022), a total of 550 adolescents were targeted for recruitment to ensure sufficient power for both univariate and multivariable analyses.

### Statistical analysis

Descriptive statistics summarised participant characteristics and survey responses. Mann–Whitney *U* tests were used to analyse differences in WSL annoyance scores between sexes and between participants who had previously seen WSLs and those who had not. The Kruskal–Wallis test was used to analyse differences in annoyance scores across treatment durations and education levels of the participants and their mothers. Spearman’s rank correlation (*ρ*) was used to analyse the association between WSL annoyance scores, adherence to CPGs, and age. Non-parametric tests were applied due to the non-normal distribution of annoyance and adherence scores. All statistical analyses were performed using SPSS v28 (IBM Corp., Armonk, NY, USA), with statistical significance set at *p* ≤ 0.05.

## Results

### Sample characteristics

**Fig. 2:**
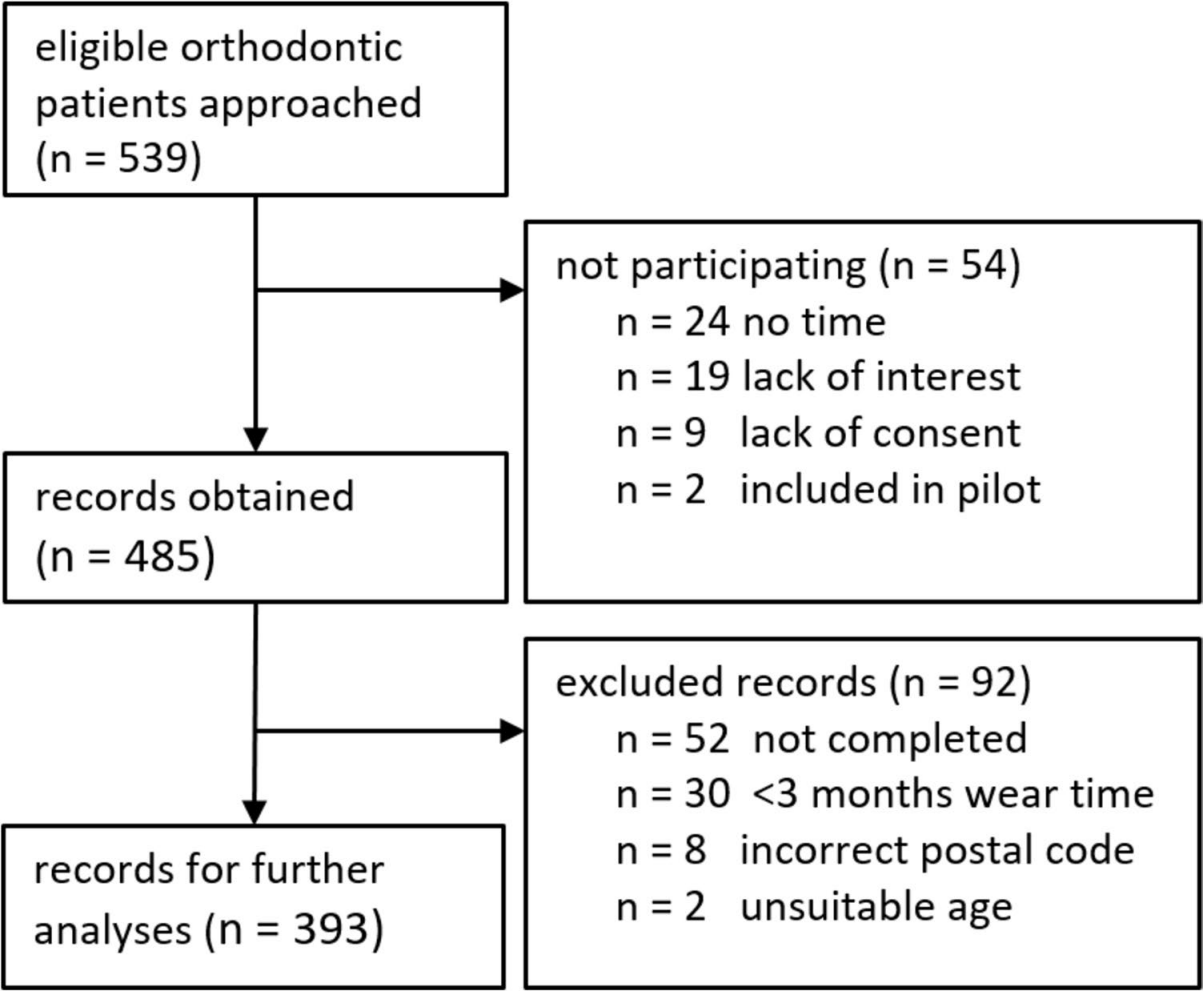
Flow diagram of participant recruitment and inclusion. A total of 539 patients were approached; 393 were included in the analysis. Reasons for exclusion and non-participation are detailed

A total of 393 of 539 eligible adolescent patients were included in the survey (Fig. 2 and Table 1). Additional exclusions resulted from incomplete questionnaires or failure to meet predefined inclusion criteria. A majority were female (57.3%), with the most common ages being 13 (27.5%) and 14 years (21.4%). Most participants were enrolled in either middle (44.6%) or high (45.6%) education. More than half (57.3%) had been in treatment with buccal fixed appliances for at least 12 months. Approximately one-third (37.7%) confirmed having seen an image of WSLs previously (Table 2).

**Table 1:** Participant characteristics of the sample (*n* = 393)

Participant characteristics Total, *n* = 393	Frequency, *n* (%)	
**Sex**		
Male	157	(39.9%)
Female	225	(57.3%)
Undisclosed/different	11	(2.8%)
Age*		
12	64	(16.3%)
13	108	(27.5%)
14	84	(21.4%)
15	56	(14.2%)
16	53	(13.5%)
17	28	(7.1%)
**Current education-level participant**		
Low education	19	(4.8%)
Middle education	175	(44.6%)
High education	179	(45.6%)
Missing/different	20	(5.1%)
**Completed education-level mother**		
Low education	2	(2.3%)
Middle education	115	(29.3%)
High education	170	(43.3%)
Missing/different	99	(25.2%)
**Months in orthodontic treatment**		
3 to < 6 months	57	(14.5%)
6 to < 12 months	111	(28.2%)
≥ 12 months	225	(57.3%)

Adapted from van Doornik et al. 2025

*Age measured in years (12–17)

**Table 2:** Associations between participant characteristics and White Spot Lesions (WSLs) annoyance

Participant characteristics Total, *n* = 393	Frequency, *n* (%)		WSL Annoyance score Median (IQR)	*p* value	Statistical test
**WSL annoyance sum score**	393	(100%)	13.0 (11.0,14.0)	n.a	n.a
3	1	(0.3%)			
6	2	(0.5%)			
7	3	(0.8%)			
8	11	(2.8%)			
9	29	(7.4%)			
10	31	(7.9%)			
11	46	(11.7%)			
12	71	(18.1%)			
13	54	(13.7%)			
14	50	(12.7%)			
15	95	(24.2%)			
**Seen WSL Image before**					
Yes	148	(37.7%)	13.0 (11.3, 15.0)	**0.004**	MWU
No	181	(46.1%)	12.0 (11.0, 14.0)		
I don’t know/missing	64	(16.3%)	12.0 (11.0, 14.0)		
Sex*				0.362	MWU
Male	157	(39.9%)	12 (10, 14)		
Female	225	(57.3%)	13 (11, 14)		
Undisclosed/different	11	(2.8%)			
Age**				0.343	0.048 SC
12	64	(16.3%)	13.0 (11.0, 15.0)		
13	108	(27.5%)	12.0 (11.0, 14.0)		
14	84	(21.4%)	12.0 (11.0, 14.0)		
15	56	(14.2%)	13.0 (10.0, 15.0)		
16	53	(13.5%)	14.0 (12.0, 15.0)		
17	28	(7.1%)	13.0 (11.0, 15.0)		
**Months in orthodontic treatment**				0.869	KW
3 to < 6 months	57	(14.5%)	12.0 (11.0, 15.0)		
6 to < 12 months	111	(28.2%)	12.0 (10.0, 14.0)		
≥ 12 months	225	(57.3%)	13.0 (11.0, 14.0)		
Current education-level participant**				0.878	KW
Low education	19	(4.8%)	13.0 (11.0, 15.0)		
Middle education	175	(44.6%)	13.0 (11.0, 14.0)		
High education	179	(45.6%)	13.0 (11.0, 15.0)		
Missing/different	20	(5.1%)			
Completed education-level mother**				0.066	KW
Low education	2	(0.5%)	11.0 (8.5, 12.5)		
Middle education	115	(29.3%)	12.0 (11.0, 15.0)		
High education	170	(43.3%)	13.0 (11.0, 14.0)		
Missing/different	99	(25.2%)			
Adherence sum score***				**0.003**	0.149 SC
1	4	(1.0%)	14.5 (13.3, 15.0)		
2	9	(2.3%)	11.0 (8.5, 13.0)		
3	40	(10.2%)	12.5 (11.0, 14.8)		
4	106	(27.0%)	12.0 (10.0, 14.0)		
5	145	(36.9%)	13.0 (11.0, 15.0)		
6	89	(22.6%)	13.0 (12.0, 14.5)		

Adapted from van Doornik et al. 2025. Statistical tests were applied to analyse differences between the groups with regard to Annoyance sum score *MWU*; Mann–Whitney *U* test, *KW*: Kruskal–Wallis test, *SC*: Spearman’s correlation

*comparison between boys and girls

**Age measured in years (12–17), ** comparison between low, middle, and high education

***total points participants meet the ACP guideline, n.a., not applicable

### Annoyance score

The median WSL annoyance score was 13 (IQR: 11 to 14) (Table 2). Notably, 24.2% of respondents reported the maximum score of 15, reflecting negative perceptions regarding WSLs.

### Associations with WSL awareness and participant characteristics

Participants who had previously seen a WSL image reported significantly higher annoyance scores than those without prior awareness (*p* = 0.004) (Table 2). Annoyance scores did not differ significantly by sex (*p* = 0.362) or age groups (no trend across ages 12–17 y, Spearman’s *ρ* = 0.048, *p* = 0.343). Treatment duration (*p* = 0.869), participant educational level (*p* = 0.878), and maternal education level (*p* = 0.066) were not associated with annoyance scores.

### Adherence to clinical practice guidelines

A weak but statistically significant positive association was observed between WSL annoyance scores and higher adherence to CPGs (Spearman’s *ρ* = 0.149, *p* = 0.003) (Table 2). In contrast, WSL awareness was not significantly associated with adherence score (Mann–Whitney *U* = 13,591.5, *p* = 0.810). The median adherence score was 4 out of 6 (IQR: 3 to 5) in both groups (those who had and had not seen a WSL image previously).

### Attitudes towards white spot lesions

WSLs were generally perceived as undesirable (Table 3). A large proportion of participants (86.2%) agreed or completely agreed that developing WSLs, whilst wearing brackets would be annoying. Similarly, 73.3% expressed annoyance at the idea of WSLs being visible after treatment, 76.8% indicated that they would seek treatment if WSLs were present (Table 3), and 65.9% believed that WSLs would become less severe with good oral hygiene after bracket removal.

**Table 3:** Participant responses on perceived White Spot Lesions (WSLs) annoyance, knowledge of WSL remineralisation, and willingness to seek treatment

Statement Total, *n* = 393	Completely disagree *n* (%)	Disagree, *n* (%)	Nor disagree, nor agree, *n* (%)	Agree, *n* (%)	Completely agree, *n* (%)
I would find it annoying if I get WSLs whilst I have brackets*	5 (1.3%)	6 (1.5%)	43 (10.9%)	144 (36.6%)	195 (49.6%)
I would find it annoying if my brackets have to come off early due to WSLs*	12 (3.1%)	23 (5.9%)	80 (20.4%)	136 (34.6%)	142 (36.1%)
I find it annoying that others can see I have WSLs when the brackets are off*	12 (3.1%)	21 (5.3%)	72 (18.3%)	140 (35.6%)	148 (37.7%)
I think WSLs get less severe if I brush well after brackets are removed	11 (2.8%)	26 (6.6%)	97 (24.7%)	182 (46.3%)	77 (19.6%)
If I get WSLs, I would seek treatment for them	3 (0.8%)	15 (3.8%)	73 (18.6%)	149 (37.9%)	153 (38.9%)

Frequency and percentages, answers on a 5-point Likert scale

*Annoyance sum score was calculated as a sum of the individual statements, resulting in a score ranging from 3 to 15

## Discussion

The present study investigated whether adolescents undergoing fixed appliance therapy were aware of WSLs, and whether their perceived annoyance due to WSLs was associated with adherence to CPGs for WSL prevention during treatment. The study’s hypothesis was only partially supported. Prior awareness of WSLs was significantly associated with higher reported annoyance. However, this increased awareness did not translate into better adherence to recommended caries preventive behaviours. The association found was very weak, with an explained variance (ρ^2^) of 2%. This small-effect size is unlikely to be of any clinical relevance. No statistically significant associations were found between WSL annoyance and participant characteristics (age, sex, education level, or treatment duration). Maternal educational level, used as a proxy for socioeconomic and health literacy background, approached statistical significance (*p* = 0.066), suggesting a possible trend whereby higher maternal education was associated with greater annoyance regarding WSLs. Although exploratory, this trend suggests that parental educational background may influence adolescents’ perceived annoyance regarding WSLs.

In the Netherlands, caries prevention in orthodontic patients is addressed through a combination of two national clinical practice guidelines. The general ‘Advice on Caries Prevention’, issued by the Ivoren Kruis (the Dutch national institute for oral health prevention), applies to all dental patients, including those undergoing orthodontic treatment, and is complemented by the orthodontic-specific ‘White Spot Lesion guideline’ issued by the Dutch Association of Orthodontist (IvorenKruis 2011; NVvO 2022). Together, these guidelines form the framework for caries prevention during orthodontic treatment and encompass recommendations on oral hygiene practices, fluoride use, dietary habits, interdental cleaning, and regular professional dental follow-up.

Most participants indicated that they would find WSLs bothersome. Despite 76.8% expressing a willingness to have WSLs treated after orthodontic treatment, adherence to CPGs remained suboptimal. These findings are consistent with previously reported results from the same study cohort, which revealed that only 22.6% of adolescents adhered to all six recommended CPGs (van Doornik et al. 2025). Importantly, only 37.7% of participants reported having previously seen an image of a WSL, indicating that most patients may have limited awareness of the increased presence and clinical appearance of WSLs. This highlights a potential opportunity to incorporate visual aids into patient oral hygiene enhancement and caries prevention strategies.

Although many participants reported high aesthetic concern regarding WSLs, this did not appear to translate into sustained improvements in CPG adherence, suggesting that appearance-related concern may raise awareness but is insufficient to drive lasting behavioural change without additional knowledge, skills, and support. Essential factors, such as confidence in the effectiveness of caries preventive actions, perceived ability to act consistently, and social reinforcement, may be critical. Previous research supports this view, showing that aesthetic motivation alone seldom sustains improved oral hygiene and dietary behaviours without structured feedback, reinforcement, or personalised guidance (Ghaffari et al. 2018; Asimakopoulou and Newton 2015; Bilici Gecer and Dursun 2024). These findings highlight the need for multifactorial interventions that address both motivation and contextual and psychological factors required for sustained adherence.

Earlier research has suggested that psychosocial factors, such as self-efficacy, intention, and planning, may influence oral health behaviours in adolescents (Scheerman et al. 2016), although consistent behavioural change remains difficult to achieve. The present findings indicate that annoyance alone is not a strong behavioural driver.

Evidence from a recent umbrella review (Yu et al. 2025) supports this interpretation. Amongst 24 systematic reviews, only topical fluoride applications, reminder systems, and sealants were consistently effective in preventing WSLs. Other widely used measures, such as crèmes containing casein phosphopeptide–amorphous calcium phosphate, fluoride-releasing bonding agents, or laser treatments, showed insufficient or inconsistent evidence. Notably, reminder systems, whether delivered via app, text message, or physical prompts, were amongst the few interventions with consistently positive behavioural outcomes. It was clear, based on the outcomes of that review, that these interventions succeed only when repeated, are contextually integrated with visual and interactive feedback, and tailored to adolescent needs. These insights are consistent with the interpretation that, whilst aesthetic concern or awareness may create initial motivation, sustained adherence is more likely facilitated by structured and repeated reminders.

The current findings also prompt reflection on the delivery of CPGs; the impact of guidelines depends not only on their content but also on how they are delivered; repetition, personalisation, and integration into daily clinical routines are essential for adherence (Asimakopoulou and Newton 2015).

Interestingly, the perception of WSLs as undesirable was consistently independent of participant characteristics in the present study. Earlier research suggested that patients with more severe malocclusions may be more willing to tolerate such side-effects if functional improvement is achieved (Silvola et al. 2014). More recent evidence indicates that orthodontic patients are generally more accepting of WSLs than oral health professionals, whilst higher education and better oral hygiene are associated with more critical views (Huang et al. 2024). In the current study, however, this pattern was not observed, and the severity of WSLs was not assessed. These findings suggest that patients’ perspectives on WSLs differ from those of professionals and warrant further investigation.

A notable finding was the high willingness amongst adolescents to seek treatment for WSLs, combined with the common belief that these lesions would become less severe after appliance removal if oral hygiene was improved. This likely reflects aesthetic concern rather than informed awareness of evidence-based treatment options, which remain limited. Although WSLs may show partial improvement after appliance removal with adequate oral hygiene and fluoride exposure, the expectation of complete resolution is often unrealistic and has been reported in earlier studies (Maxfield et al. 2012; Ludovichetti et al. 2025). Although WSLs may show partial remineralisation with good oral hygiene and fluoride exposure, their aesthetic appearance often remains unsatisfactory due to persistent enamel opacity and may take a long time to improve (Shungin et al. 2010; Ludovichetti et al. 2025; Ogaard et al. 1988). Management options for WSLs, such remineralisation or resin infiltration, can improve aesthetics but often to a limited degree and may involve considerable costs and chair time (Khoroushi and Kachuie 2017; Ludovichetti et al. 2025). Resin infiltration, however, has emerged as a minimally invasive option that appears more effective in masking WSLs than fluoride varnish or natural remineralisation, with outcomes that remain stable for several years (Bourouni et al. 2021; Kashash et al. 2024; Prada et al. 2024). These findings emphasise the need for clearer patient education on the irreversible nature of WSLs and the importance of preventive care during orthodontic treatment.

Given the relatively large sample size, the present study offers insight into behavioural trends within the adolescent orthodontic population. To improve adherence, we recommend a targeted and evidence-informed strategy for WSL prevention. Potential strategies include visual aids, repeated communication, app-based reminders, and motivational interviewing, with an emphasis on behaviourally informed, visually reinforced, and longitudinally integrated approaches that go beyond passive information and are more likely to support sustained adherence.

## Limitations

The cross-sectional design limits the ability to infer any causality between WSL awareness, annoyance, and adherence. Self-reported data may have introduced recall and social desirability bias, particularly with respect to oral hygiene behaviours and recollection of information provided by the orthodontist. Although the presence of a researcher during data collection likely reduced misunderstandings—especially for questions concerning dietary habits, maternal education, and fluoride use—these biases cannot be fully excluded. Whilst a statistically significant association was observed between perceived annoyance and adherence, this association was weak and explained only a small proportion (2%) of the variance. Given this limited effect size and the exploratory nature of the analysis, additional multivariable modelling was deemed not meaningful.

Furthermore, this study did not assess how CPGs were translated into practice-level protocols and how or whether CPGs were delivered to patients, which may influence adherence. As all participants received privately financed orthodontic care, in accordance with the inclusion and exclusion criteria of the present study and the Dutch orthodontic care setting, differences in adherence between publicly funded and private care settings could not be explored. Finally, as the sample was drawn from one region in the Netherlands, findings may not be generalisable to other healthcare settings or cultures.

Whilst the effectiveness of current patient education was not directly assessed in this study, the limited behavioural impact observed highlights the need to critically reconsider how caries preventive recommendations are communicated and supported in daily practice.

## Conclusions

Although adolescents undergoing fixed appliance therapy expressed concern about WSLs and a strong willingness to seek treatment if they occurred, this concern did not appear to translate into improved adherence to CPGs. Neither prior awareness of WSLs nor higher levels of perceived annoyance were meaningfully associated with better caries preventive behaviours. These findings suggest that motivation or concern alone may be insufficient to drive CPG adherence during orthodontic treatment.

## Ethical approval

The Medical Ethics Review Board of the UMCG (METc 2023/014) reviewed and approved the study, determining that it was not subject to the Dutch Medical Research Involving Human Subjects Act (WMO). Written informed consent was obtained from all participants and their legal guardians prior to participation.

## Data Availability

All data supporting the findings of this study are available from the corresponding author upon reasonable request.
